# Genome-wide association studies on HIV susceptibility, pathogenesis and pharmacogenomics

**DOI:** 10.1186/1742-4690-9-70

**Published:** 2012-08-24

**Authors:** Daniëlle van Manen, Angélique B van ‘t Wout, Hanneke Schuitemaker

**Affiliations:** 1 Department of Experimental Immunology, Sanquin Research, Landsteiner Laboratory, and Center for Infectious Diseases and Immunity Amsterdam (CINIMA), Academic Medical Center, University of Amsterdam, Meibergdreef 15, 1105 AZ, Amsterdam, The Netherlands; 2 Crucell Holland B.V., Archimedesweg 4-6, 2333 CN, Leiden, The Netherlands

**Keywords:** Genome-wide association studies, Single-nucleotide polymorphisms, Host genetics, HIV susceptibility, HIV pathogenesis

## Abstract

Susceptibility to HIV-1 and the clinical course after infection show a substantial heterogeneity between individuals. Part of this variability can be attributed to host genetic variation. Initial candidate gene studies have revealed interesting host factors that influence HIV infection, replication and pathogenesis. Recently, genome-wide association studies (GWAS) were utilized for unbiased searches at a genome-wide level to discover novel genetic factors and pathways involved in HIV-1 infection. This review gives an overview of findings from the GWAS performed on HIV infection, within different cohorts, with variable patient and phenotype selection. Furthermore, novel techniques and strategies in research that might contribute to the complete understanding of virus-host interactions and its role on the pathogenesis of HIV infection are discussed.

## Introduction

There is considerable heterogeneity in HIV-1 susceptibility and in disease progression rates after infection. Certain people are relatively resistant to HIV-1 infection and remain uninfected despite multiple exposures to HIV-1, while others are infected upon first exposure. After seroconversion, some individuals progress to AIDS in as little as 2 years, while others remain symptom-free for more than 15 years. This variation between individuals is determined by both viral and host factors.

The emergence of HIV-1 variants that use coreceptor CXCR4 rather than CCR5 in the course of infection is associated with an accelerated CD4^+^ T-cell decline and more rapid progression to AIDS [1,2]. Other evidence that viral factors may influence the clinical course of HIV-1 infection comes from a cohort of long-term nonprogressors (LTNPs) who were all infected with an HIV-1 variant that was attenuated due to a deletion in the viral nef gene [3].

The first polymorphisms in host genetic factors that affected HIV-1 infection and disease were determined using candidate gene studies, in which genetic variants of host factors that were already known or suspected to play a role in HIV-1 pathogenesis and immune regulation were tested for association with HIV-1 infection and/or disease progression. These studies identified several important host polymorphisms associated with HIV-1 infection and pathogenesis [4-14]. The human leukocyte antigen (HLA) type is a strong example of a host factor that is associated with HIV-1 disease course. HLA-B*5701 and HLA-B27 are more prevalent among LTNPs whereas HLA-B35 is associated with an accelerated progression to AIDS [15-17]. Another important host factor polymorphism is a 32 basepair deletion in CCR5 (CCR5Δ32), the major coreceptor for HIV-1. This deletion, which results in a truncated protein product that is no longer expressed on the cell surface, provided nearly complete protection against HIV-1 infection in individuals homozygous for this deletion [18-20]. Individuals carrying the heterozygous CCR5Δ32 genotype have sufficient CCR5 expression on the cell surface to support infection; however, this heterozygous genotype is associated with delayed disease progression after HIV-1 infection [18,21,22].

In the case of CCR5, the association between the genetic polymorphism and disease progression has even resulted in the development of new antiviral strategies to block CCR5 in HIV-1 infected individuals [23]. These developments illustrate the potential of host genetic research to combat HIV-1 infection and AIDS. However, even when combined, these genetic variations together still only explained a small fraction of the variability of HIV-1 control between individuals.

The more recent genome-wide association studies (GWAS) offer a hypothesis-free analysis to scan the complete human genome for additional factors without *a priori* knowledge about their role in complex diseases. Following the completion of the human genome sequence in 2003 [24], the HapMap project was launched [25] in which commonly occurring genetic variations were identified along the complete genome and across several populations. These mostly single DNA mutations were called single-nucleotide polymorphisms (SNPs). In most of the genome, combinations of SNPs appear to be found together in blocks with strong linkage disequilibrium (LD), which gives the opportunity to cover almost the whole genome on the chips used in GWAS, by selecting “tagSNPs” that represent the LD blocks [26]. In this review, we will discuss GWAS performed on HIV-1, focusing on the differences in cohorts and phenotypes used.

## Review

### Genome-wide association studies on HIV-1

#### Host genetic factors that associate with viral load control

The first reported GWAS was performed on 486 individuals from the Euro-CHAVI cohort, and used HIV RNA viral load at set point as phenotype, which is known to be predictive for disease progression. (see Table 1 for an overview of all published GWAS up to date) [27]. In the association analysis, using linear regression, two loci were genome-wide significantly associated with viral load at set point. Without an *a priori* hypothesis, a stringent correction for multiple tests in GWAS is required to avoid false-positive errors. The current standard for genome-wide significance in GWAS is a P-value below 5x10^-8^. One of these loci is tagged by SNP rs2395029 near the HLA complex 5 gene (HCP5), a gene that is localized within the MHC class I region. SNP rs2395029 is in nearly absolute LD with HLA-B*57, which was already known to be protective against disease progression as described above. The other SNP, rs9264942, is located 35kb from the HLA-C gene. It was shown that the variation within the 3’ UTR region of HLA-C regulates binding of the microRNA hsa-miR-148 to its target site, resulting in differential expression of the HLA-C gene [28,29]. Despite the fact that the HCP5 SNP and −35 HLA-C SNP were in moderate LD, Fellay *et al*. showed an independent effect of each of these variants on the viral load set point variation between individuals. This first GWAS additionally identified a set of seven SNPs that were in high LD and located close to ring finger protein 39 (RNF39) and zinc ribbon domain-containing protein 1 (ZNRD1), to be associated with progression to CD4^+^ T cell count below 350 cells/ml [27]. The replication study by Catano *et al*. [30] showed that the causal effect of these SNPs on HIV-1 disease progression might be because of a very strong LD between these SNPs and HLA-A10.

**Table 1:** Description of GWAS on HIV infection and the most interesting signals discovered

**cohort**	**year**	**Phenotype**	**Most important association**	**P-value**	**Validation**	**ethnicity**	**ref**
CHAVI	2007	RNA VL setpoint	HCP5 (rs2395029)	9.4E-12	Significant, confirmed	Caucasian	[27]
			−35 HLA-C (rs9264942)	3.8E-09	Significant, confirmed		
		CD4 T cell decline	ZNRD1 (rs9261174)	3.9E-07	Confirmed		
ANRS PRIMO	2008	plasma HIV RNA primary infection	HCP5 (rs2395029)	9.3E-07	Significant, confirmed	Caucasian	[31]
		cellular HIV DNA primary infection	HCP5 (rs2395029)	6.7E-07	Significant, confirmed		
GRIV	2009	long-term nonprogression	HCP5 (rs2395029)	6.8E-10	Significant, confirmed	Caucasian	[32]
			C6orf48 (rs9368699)	5.3E-07	Putative		
Euro-CHAVI, MACS	2009	RNA VL setpoint	HCP5 (rs2395029)	4.5E-35	Significant, confirmed	Caucasian	[33]
			−35 HLA-C (rs9264942)	5.9E-32	Significant, confirmed		
GRIV	2009	rapid progression	PRMT6 (rs4118325)	6.1E-07	Putative	Caucasian	[34]
			SOX5 (rs1522232)	1.8E-06	Putative		
International HIV controllers study	2010	VL controllers	>300 SNPs in MHC	<5.0E-08	Significant	Caucasian, African, Hispanic	[35]
			−35 HLA-C (rs9264942)	2.8E-35	Significant, confirmed		
			HCP5 (rs2395029)	9.7E-26	Significant, confirmed		
			MICA (rs4418214)	1.4E-34	Significant		
			PSORSIC3 (rs3131018)	4.2E-16	Significant		
Queen Elizabeth Central Hospital, Malawi	2010	mother-to-child transmission	HS3ST3A1 (rs8069770)	3.8E-05	Putative	African	[36]
MACS	2010	progression to AIDS	PROX1 (rs17762192)	6.2E-07	Confirmed	Caucasian	[37]
DoD HIV NHS and MACS	2010	RNA VL setpoint	HLA-B*5703	5.6E-10	Significant, confirmed	African	[38]
GRIV, MACS, ACS	2010	long-term nonprogression (VL > 100 cp/ml)	CXCR6 (rs2234358)	9.7E-10	Significant, confirmed	Caucasian	[39]
CHAVI, Malawi	2011	HIV acquisition				African	[40]
MACS	2011	progression to AIDS 1987	PARD3B (rs11884476)	3.4E-09	Significant, confirmed	Caucasian, African	[41]
blood donors, Sanquin Amsterdam	2011	*in vitro* HIV-1 replication in macrophages	DYRK1A (rs12483205)	2.2E-05	Putative	Caucasian	[42]
ACS	2011	progression to AIDS, or AIDS-related death	AGR3 (rs152363)	3.5E-06	Putative	Caucasian	[43]
Thailand	2011	Nevaripine tolerance	CCHCR1 (rs1265112)	1.2E-08	Significant	Asian	[44]
African serodiscordant couples cohort	2011	HIV acquisition				African	[45]

VL; viral load.

In a follow-up study, Fellay *et al*. [33] performed a GWAS on an extended population (n = 2362) to identify additional genetic variants that could explain the variability of HIV-1 control between individuals. As expected, this study confirmed the association of the HCP5 SNP (rs2395029, P = 4.5 x 10^-35^) and the −35 HLA-C SNP (rs9264942, P = 5.9 x 10^-32^) with viral load at set point. Next to these already known variants, the authors identified other, independent loci in the MHC that were associated with viral load control. For example rs9468692, located in the 3’ region of the TRIM10 gene, and the non-synonymous coding SNP rs8192591, located in the 9^th^ exon of the NOTCH4 gene.

Dalmasso *et al*. [31] also used viral load as a disease phenotype in their GWAS, but evaluated plasma HIV-RNA during primary infection rather than at set point. This study analyzed 605 seroconverters in a case–control study, comparing 45 long-term HIV controllers, with an RNA viral load below 400 copies/ml for more than 10 years, with the rest of the HIV-infected individuals. The protective allele of SNP rs10484554, located in the region between HLA-C and HLA-B, was genome-wide significantly (P = 3.58 x 10^-9^) over-represented among the long-term HIV controllers. These authors identified HCP5 rs2395029 to be most strongly associated with HIV-1 DNA levels in the first patient samples drawn at the time of enrolment during primary infection (P = 6.72 x 10^-7^). These HIV-1 DNA levels were considered as a marker of the HIV reservoir in their seroconverters. This SNP was also associated with HIV controller status, thereby confirming the results from the first GWAS by Fellay *et al*.

Viral load was also used as the phenotype in the multinational HIV Controllers study [35]. This large cohort of HIV-infected individuals was divided into elite and viremic controllers (n = 1526), which are seropositive individuals who are able to control viral load to levels below 50 or 2,000 copies of viral RNA/ml plasma respectively, and HIV-1 progressors (n = 2648), who failed to ever control viremia without therapy. Over 300 SNPs were identified to be genome-wide significantly associated with viral load (P < 5.0 x 10^-8^), and all were located within the MHC gene region on chromosome 6. Only four of these SNPs were independently associated with viremic control: the already known SNPs −35 HLA-C (P = 2.8 x 10^-35^) and HCP5 rs2395029 (P = 9.7 x 10^-26^), and two novel SNPs, rs4418214, located near MICA (P = 1.4 x 10^-34^), and rs3131018 in PSORSIC3 (P = 4.2 x 10^-16^), a gene that has been implicated in psoriasis. Interestingly, the authors identified several specific amino acids in the HLA-B peptide binding groove to be even more strongly associated with viral load control than any SNP found in the GWAS, or any of the HLA alleles.

#### Host genetic factors that associate with HIV-1 disease progression

Two GWAS were performed in the Genomics of Resistance to Immunodeficiency Virus (GRIV) cohort to look for genetic associations with extreme phenotypes in HIV-1 infection in either LTNPs [32] or rapid progressors (RP) [34]. The nonprogression GRIV GWAS compared 275 LTNPs to a control group of 1352 seronegative individuals and found HCP5 rs2395029 to be most strongly associated with nonprogression after HIV-1 infection (P = 6.8 x 10^-10^). Mainly associations with genetic variation in chromosome 6 were found and this GWAS again confirmed association between HIV-1 control and the HCP5 and the ZNRD1 locus identified by the EURO-CHAVI cohort. More recently, this GWAS was reanalyzed to specifically identify genetic variants that associate with LTNP without elite control of the viral load [39]. To this end, the authors compared 697 uninfected individuals with 186 LTNPs, excluding elite controller patients with a viral load below 100 copies/ml. SNP rs2234358 in the CXCR6 gene was identified to be associated with LTNP and this association could be replicated in three independent European studies (P = 9.7 x 10^-10^).

The only genome-wide analysis of RP to date [34] revealed several interesting loci outside the MHC region in a case–control study of 85 HIV-1-infected patients who had experienced rapid disease progression and who were compared with 1352 seronegative individuals. SNPs rs4118325 (P = 6.1 x 10^-7^), in the vicinity of PRMT6 and rs1522232 in SOX5 (P = 1.8 x 10^-6^) were amongst the top SNPs that were associated with rapid progression after HIV-1 infection. These associations were, however, not significant after correction for multiple testing. The exact potential mechanism of action for these two SNPs is unknown. Although analysis of RP yields unique loci, these individuals are underrepresented in most cohorts. This low number of RP could be an explanation for the lack of genome-wide significant signals, and indicates the difficulty of replicating signals in other RP cohorts.

A multi-stage GWAS in US seroconverters compared RP (n = 51), moderate progressors (n = 57) and LTNPs (n = 48) [37]. Genetic variation rs17762192, upstream of PROX1, a negative regulator of IFN-γ expression in T cells [46], was associated with slower progression to AIDS (P = 6.2 x 10^-7^). Although this SNP upstream of PROX1 was not genome-wide significantly associated with slower progression to AIDS, the loci could be replicated in an independent population of 590 HIV-infected seroconverters.

Troyer *et al*. [41] identified a cluster of SNPs in the gene PARD3B to be associated with a delayed survival time to clinical AIDS (P = 3.4 x 10^-9^) in a GWAS amongst US seroconverters (n = 755). One of the PARD3B variants in this cluster could be confirmed in two European cohorts of rapid progressors. PARD3B interacts with members of the SMAD family, which are known to interact directly with HIV-1 [47]. The HCP5 rs2395029 signal was not found to be associated with survival time to clinical AIDS in this cohort.

Another GWAS on HIV-1 disease progression was performed in the Amsterdam Cohort Studies (ACS) [43]. In this study in 404 HIV-infected individuals, the association of SNPs with survival time to AIDS-diagnosis and AIDS-related death was tested. Albeit not genome-wide significant, SNP rs152363 showed a tendency to association with disease progression after HIV-1 infection in both the ACS and the GRIV cohort (P = 3.5 x 10^-6^). Furthermore, this GWAS showed that in the ACS the HCP5 rs2395029 was also significantly associated with delayed progression to AIDS and AIDS-related death, although the effect was notably reduced when viral load at set point was included as a covariate in multivariate analysis [48].

Results from GWAS on disease progression show that replication in cohorts with alternative phenotypes has proven to be challenging. Moreover, not many prospective seroconversion cohorts exist that have sufficient follow up time between the moment of HIV-1 infection and disease progression, which complicates these survival studies.

#### GWAS in African populations

Human genetic studies in disease in general have been focusing almost exclusively on individuals of European ancestry. Until 2011 more than 75% of studies in the catalog of published GWAS were analyzing individuals from European ancestry [49]. Remarkably, less than 5% of the GWAS were focusing on individuals from African descent. For HIV studies this is discouraging, since the HIV-infection prevalence rate is particularly alarming in sub-Saharan Africa (http://unaids.org/globalreport). An obvious reason for this inequality in population coverage in GWAS is the availability of study populations with DNA samples and documented phenotypes. In addition, African populations are characterized by lower levels of LD [50], which has led to poor coverage of the whole-genome by proxy SNPs on the initial chips used in GWAS. Because of this uneven prevalence of European ancestry in GWAS, and the different genetic make-up of other populations, reflected in differences in HLA class I allele frequencies and in LD between populations, associations of genetic variations that were identified in Caucasian HIV-infected individuals can be absent from other populations. Indeed HCP5 rs2395029 was not found to be associated with viral load at set point in an African population [51].

The first published GWAS in a non-European population searched for associations with more than 500,000 SNPs with viral load at set point in 515 African Americans [38]. Although no loci were genome-wide significantly associated with viral load at set point, one of the strongest associations was a SNP tagging the HLA-B*5703 allele. Individuals carrying the HLA-B*5703 allele, have a significantly lower viral load at set point (P = 5.6 x 10^-10^), thereby again emphasizing the important association between HLA-B*57 and the control of viral load after HIV infection, both in individuals of African and European ancestry.

In sub-Saharan Africa, children are infected with HIV predominantly through mother-to-child transmission (MTCT). Joubert *et al*. [36] conducted a GWAS in a Malawi cohort to identify the genetic host factors associated with vertical transmission of HIV. In this study, HIV-negative (n = 126) and positive (n = 100) children from HIV-infected mothers, were compared. The top signal (rs8069770) was not significant after correction for multiple testing (P = 3.79 x 10^-5^). This could be due to limited sample size, causing the study only to be powered to detect large genetic effects. SNP rs8069770 is located within HS3ST3A1, a gene involved in heparin sulfate biosynthesis, which interestingly is very abundantly expressed in the placenta. In another population of individuals from Malawi, a country with high prevalence of HIV-1 infection, a GWAS was performed to identify host determinants of HIV-1 susceptibility [40]. Unfortunately, no single SNP yielded a significant P-value after correction for multiple testing, when the authors compared 848 high-risk seronegatives with 531 HIV-1 seropositive individuals. Failing to detect a genome-wide significant signal could be due to the difficulty to quantify the level of HIV-1 exposure in this population. Although the studied individuals are assumed to be in a high-risk category as they are attending STI clinics in a region with a high HIV-1 prevalence and incidence, there were no actual data collected on exposure details (e.g. number of partners and type of sexual contacts). Furthermore, HIV-1 susceptibility can be strongly influenced by other factors, like circumcision status of male partner, concurrent STIs and viral load level of the donor.

A recent GWAS selected participants from two cohorts of African HIV-1 serodiscordant heterosexual couples [45], thereby increasing the probability that the HIV-1 negative partners have a risk for HIV-1 acquisition. In this study 496 HIV-1 infected individuals were compared to 302 matched HIV-1 uninfected individuals with similar documented HIV-1 exposure. Nevertheless, after correction for multiple testing, no SNPs were significantly associated with HIV-1 susceptibility, or with viral load at set point among the subset of HIV-1 infected participants.

#### Alternative phenotypes in GWAS

Most GWAS on HIV infection have focused on viral load control and disease progression. However, alternative phenotypes might be interesting to identify additional host genetic factors for HIV replication and pharmacogenomics.

We performed a genetic association analysis on *in vitro* HIV-1 susceptibility of primary monocyte-derived macrophages [42]. The top signal identified in this study, while not genome-wide significant, was rs12483205, a SNP intronic of DYRK1A (P = 2.2 x 10^-5^). This SNP appeared to be associated with HIV-1 disease progression as well in two independent cohort studies.

The use of antiretroviral drugs has been associated with severe toxicities, including hypersensitivity reactions, neurotoxicity and liver damage [52,53]. Several candidate gene studies on the association between genetic determinants of people infected with HIV-1 and clinical toxicity resulting from different antiretroviral drugs have been performed. The study by Chantarangsu *et al*. [44] was the first to conduct a GWAS on therapy-side effects in HIV-infected patients. The study was performed in 72 HIV-infected Thai patients with nevaripine-induced rash, compared with 77 nevaripine-tolerant patients, and candidate genes were followed-up in an additional group of patients. SNPs rs1265112 and rs746647, within coiled-coil a–helical rod protein1 (CCHCR1) on chromosome 6, were found to be significantly associated with susceptibility to allergic skin reactions after Nevaripine use (P = 1.2 x 10^-8^). Since the CCHCR1 gene is located close to both HLA-B and HLA-C locus, the association with rash might be explained by LD between CCHCR1 and HLA-B*3505, a previously identified genetic marker for nevaripine-induced rash [54].

## Discussion

The first GWAS on HIV infection identified variants in the HLA-region to be most dominantly associated with viral load at set point [27], which was successfully replicated in other cohorts [30-33,35], using viral load control and disease progression as phenotypes. GWAS that used clinical disease progression as a phenotype, such as LTNP, survival time to AIDS-diagnosis and AIDS-related death, identified additional genetic variants outside the HLA-region [32,34,37,39,41,43]. However, not all of these signals could be replicated in other studies and need confirmation. Multiple determinants may account for the observed variability in results from different GWAS, especially those not using viral load at set point. For example, variability in the phenotype studied, differences in the genotyping platform that was used, ancestry of the study population, gender, transmission route of infection and choices of statistical tests may influence the outcomes of these studies. Furthermore, the number of identified host factors involved in HIV infection up to now explains only a small fraction of the observed heritability. Several explanations for this “missing heritability” have been proposed such as additional common variants of small effect, low frequency variants (MAF < 5%), which have not been covered well by current GWAS, and epigenetic effects. Data from the 1000 Genomes Project (http://www.1000genomes.org) could help to identify additional associated rare variants. The 1000 Genomes Project aimed to sequence the full genome of approximately 2500 individuals, not only Caucasian individuals but also from African populations from Malawi, the Gambia and Ghana. These sequences will allow for a detailed characterization of human genome sequence variation and a further increase in sample size will likely also reveal new genetic variants.

By combining clinical data from all cohorts that have longitudinal data, a large sample set will be created which will give the opportunity to identify novel polymorphisms outside the HLA-gene region that have a small effect, a low-frequency, or a recessive association thereby explaining only a marginal portion of the observed variance. The International HIV Acquisition Consortium (IHAC) has initiated the collection of longitudinal clinical data from all cohorts that have GWAS data available [55]. Imaginably, it will be a challenge to combine all of the clinical data that were collected by the different cohorts. Other opportunities lay in more in-depth analyses of the available GWAS data, thereby focusing on pathway analysis and gene-gene interactions or by combining GWAS data with other genome-scale data sets, such as RNAi screens or gene expression profiling. These analyses may support the discovery of additional variants that did not survive the stringent multiple testing correction thresholds in the discovery studies due to limited power.

Advances in sequencing technologies will enable whole-genome sequencing (WGS) to rapidly develop and overtake the position of GWAS in genomic research. Sequencing the complete genome of cases will make it possible to capture the rare variants that might be an explanation for the missing heritability in common diseases and directly identify the causal variant.

Until WGS is inexpensive enough to be used for large sample sizes, careful selection of individuals is essential. One approach involves the selection of individuals from each side of the extremes of the phenotype distribution. For HIV-1, the sequencing of the complete genome of hemophiliacs who are known to be highly exposed to HIV-1, but who have remained uninfected has been initiated [56]. Still many technical challenges in WGS need to be overcome. Analysis techniques need to be developed in order to cope with the millions of variants that are identified per genome. Moreover, accurately coding all of the small insertions and deletions is a tremendous challenge.

Whole-exome sequencing, a strategy to selectively sequence the coding regions, can be a more cost-effective alternative to identify host genetic markers that are associated with disease. However, there are drawbacks to this technique, as only a small number of SNPs that are associated with traits are located in, or occur in, high LD with protein coding regions of genes. The vast majority of trait-associated SNPs fall in intergenic regions and noncoding introns [49,57].

The shift of genetic research from GWAS to WGS or exome sequencing might be a particularly positive development for genomics in African populations, since the causal variants are genotyped directly, without the need for high LD structures. Thereby, a smaller sample size might be needed to identify rare, causal variants, making it possible to collect smaller amounts of samples from individuals from different subpopulation structures throughout the African continent.

### Conclusions

Several host genetic determinants of HIV-1 infection and pathogenesis have been identified in the last decennia, either by the classical candidate-gene approaches or in the last four years with the help of GWAS. In all these studies, variants in the HLA-region and the coreceptor CCR5 were the most consistent and with the largest effect size. While these polymorphisms may have a large effect on the disease course in the individual, these genetic markers were only able to explain a small fraction of overall observed differences in HIV-1 infection and disease progression in the population. The missing genetic variations may be identified by combining GWAS data sets of cohorts to increase power and by looking into additional phenotypes. Furthermore, in the next couple of years the use of whole-genome sequencing will most likely allow the identification of novel rare variations that are associated with HIV-1 susceptibility and disease progression and the unraveling of pathways that are causally involved in these phenotypes. Hopefully, the ongoing genetic research may contribute substantially to the understanding of the pathogenesis of HIV-1 infection and thereby lead to the development of new strategies to combat the AIDS epidemic worldwide.

## Competing interests

The authors declare that they have no competing interests.

## Authors’ contributions

DM, AW and HS wrote the manuscript. All authors read and approved the final manuscript.
